# Peritoneal Protein Loss, Inflammation, and Nutrition: Refuting Myths

**DOI:** 10.3389/fmed.2022.884061

**Published:** 2022-05-26

**Authors:** Anabela Malho Guedes, Roberto Calças Marques, Brigitte Ribeiro, Mónica T. Fernandes, Marília Faísca, Ana Paula Silva, José Bragança, Anabela Rodrigues

**Affiliations:** ^1^Serviço de Nefrologia, Centro Hospitalar Universitário do Algarve, Faro, Portugal; ^2^Faculdade de Medicina e Ciências Biomédicas, Universidade do Algarve, Faro, Portugal; ^3^UMIB - Unidade Multidisciplinar de Investigação Biomédica, ICBAS - Instituto de Ciências Biomédicas Abel Salazar, Universidade do Porto, Porto, Portugal; ^4^Escola Superior de Saúde, Universidade do Algarve, Faro, Portugal; ^5^Algarve Biomedical Center, Universidade do Algarve, Faro, Portugal; ^6^Algarve Biomedical Center Research Institute, Universidade do Algarve, Faro, Portugal; ^7^Synlab Algarve, Faro, Portugal; ^8^Champalimaud Research Program, Champalimaud Center for the Unknown, Lisbon, Portugal; ^9^Serviço de Nefrologia, Centro Hospitalar Universitário do Porto, Porto, Portugal; ^10^ITR - Laboratory for Integrative and Translational Research in Population Health, Porto, Portugal

**Keywords:** peritoneal protein loss, peritoneal dialysis, inflammation, nutrition, overhydration

## Abstract

Peritoneal protein loss (PPL) has been correlated with mortality, malnutrition and inflammation. More recently overhydration was brought to the equation. This study aims to review classic and recent factors associated with PPL. Prevalent and incident peritoneal dialysis (PD) patients were included. Dialysate and serum IL-6 was obtained during PET. Hydration and nutritional status were assessed by bio-impedance. Linear regression and Cox regression were performed. The 78 included patients presented median values of PPL 4.8 g/24 h, serum IL-6: 5.1 pg/mL, and IL-6 appearance rate 153.5 pg/min. Mean extracellular water excess (EWexc) was 0.88 ± 0.94 L, and lean body mass index (LBMI) 17.3 ± 2.4 kg/m^2^. After mean follow-up of 33.9 ± 29.3 months, 12 patients died. Linear univariable analysis showed positive associations between PPL and small solute transport, body composition (LBMI and EWexc), comorbidities and performing CAPD (vs. cycler). PPL correlated positively with dialysate appearance rate of IL-6, but not with serum IL-6. Linear multivariable analysis confirmed positive association between PPL and EWexc (*p* = 0.012; 95%CI: 4.162–31.854), LBMI (*p* = 0.008; 95%CI: 1.720–11.219) and performing CAPD (*p* = 0.023; 95%CI: 4.375–54.190). In survival analysis, no relationship was found between mortality and PPL. Multivariable Cox regression showed Charlson Comorbidity Index (HR: 1.896, 95%CI: 1.235–2.913), overhydration (HR: 10.034, 95%CI: 1.426–70.587) and lower PPL (HR: 0.576, 95%CI: 0.339–0.978) were predictors for mortality. Overhydration, was a strong predictor of PPL, overpowering variables previously reported as determinants of PPL, namely clinical correlates of endothelial dysfunction or local inflammation. PPL were not associated with malnutrition or higher mortality, emphasizing the importance of volume overload control in PD patients.

## Introduction

The clinical significance of peritoneal protein loss (PPL) has been a matter of controversy. It has been seen as a detrimental consequence of peritoneal dialysis for many years (1, 2). Many studies correlated this protein leakage with higher mortality (3–7), malnutrition (8), and inflammation (9). Other authors found PPL to be related with cardiovascular events (10). Such conclusions are commonly explained by the concept of systemic endothelial barrier dysfunction and hence increased peritoneal leak of serum proteins could be seen a biomarker of vascular comorbidity, leading to worse survival (11). In this context, inflammation has been advocated as one of the driving forces for protein leakage. First clinical evidence arose initially from peritonitis (12), and then this debate evolved from an initial culprit systemic inflammation (9) to a consistent focus on local inflammation (11).

In spite of that, several authors have shown other survival cohorts, refuting the association with PPL with all-cause or cardiovascular mortality (10, 13–15). More recent knowledge has brought overhydration to this equation (16, 17), claiming fluid volume overload as a major culprit for PPL attributed mortality. Furthermore, other attributed consequences of PPL, such as malnutrition, have also been refuted in recent papers. Do et al. demonstrated that PPL is not associated with muscle mass, strength or sarcopenia, as long-term markers of malnutrition (18).

Controversy is far from over, and confounders should be identified. By combining critical methodological issues such as effluent and serum biomarkers, the aim of this study is to review classic and recent factors associated with peritoneal protein loss and its consequences on overall mortality in peritoneal dialysis patients.

## Materials and Methods

### Study Design, Patient Population, and Variables

This single center, longitudinal study, included prevalent and incident peritoneal dialysis, from March 2015 to March 2021, with follow up until the 31st December of 2021. Inclusion criteria comprised a modified PET, using 3.86/4.25% glucose solutions and interleukin−6 determination (both in serum and 4-h peritoneal effluent). Simultaneously, a 24-h collection of spent dialysate was obtained. Effluent protein was measured using the biuret reaction method and peritoneal protein loss (PPL) is expressed as grams per 24-h. Hydration and nutritional status were assessed by simultaneous multifrequency bioelectrical impedance (InBody S10 Body Composition Analysis; Biospace, Seoul, South Korea). Extracellular water excess (ECWexc) was calculated by ECW measured-−0.613 × intracellular water (ICW) measured (19). Exception made for overhydration control in heart failure patients (performing a daily exchange of icodextrin), all other patients were eligible for performing PET.

Baseline demographics and clinical features were registered, namely age, gender, weight, height, medication, cause of kidney failure, dialysis duration, residual renal urine volume and solute clearance. Charlson Comorbidity Index was calculated at the time of study inclusion^1^. Routine biochemical analyses were performed by an automatic chemistry analyzer (Architect ci8200 Abbot®). Creatinine was measured with an enzymatic method to prevent glucose interference. Serum high sensitivity C reactive protein (CRP) was determined by immunoturbidimetry at the time of the PET. Residual renal function (RRF) was assessed as the mean of urea and creatinine clearance from the 24-h urine collection. Urea clearance index (Kt/V urea) was derived from the 24-h urine and PD effluent collection.

All patients were treated with reduced glucose degradation products content and a normal pH dialysis solution (Baxter® and Fresenius®). The maximum glucose concentration was 2.27%/2.3%. Patients who had active infection or malignancy, experienced acute hospital admissions or a peritonitis episode during the preceding 3 months, were excluded.

### Sample IL-6 Analysis

Collected serum and peritoneal fluid samples were immediately stored at −80°C, until analysis. All samples had one or two freeze–thaw cycles before quantification. For the development of sandwich enzyme linked immunosorbent assays (ELISA), we used the DuoSet® ELISA Development System to measure Interleukin 6 (IL-6; DY206-05; R&D Systems Inc., Minneapolis, MN, USA), following the manufacturer's protocols and using all the recommended additional reagents. The ELISA was specific for human IL-6 and did not cross-react with human Recombinant human CNTF, G-CSF, gp130, IL-6 R, IL-11, IL-12, LIF, LIF R, and OSM. Duplicate readings were assayed using a TECAN Infinite®200 multimode reader (Mannedorf, Switzerland) for each standard, control, and sample and the average zero standard optical density (O.D.) was subtracted. A standard curve with the value of absorbance vs. the concentration was generated by reducing the data using the Quest Graph™ Four Parameter Logistic (4PL) Curve Calculator (20). The sample concentrations were then calculated from the determined absorbance values through the four-parameter logistic (4PL) standard curve, using the same software.

### Statistical Analysis

Shapiro-Wilk test was used to check normality of the data. Results were expressed as frequencies and percentages for categorical variables, mean ± standard deviation (SD) for continuous variables, and median (interquartile range) for skewed distributions. For description of the predictors of peritoneal protein loss uni- and multivariable linear regression were performed. Preliminary analyses were performed to ensure there was no violation of normality and linearity. All variables with a statistical association of *p* <0.2 were used to create a multiple linear regression model to determine associations with PPL. The backward method was used to choose the best model, based on the highest adjusted *R*^2^.

Survival analysis, using Cox regression, was performed firstly as univariable analysis. The conditional backward method was used in the multivariate analysis, due to the low number of events. The statistical analysis was performed using SPSS version 22.0. Statistical significance was considered at or below a 5% level.

## Results

### Patients' Characteristics

**Figure 1:**
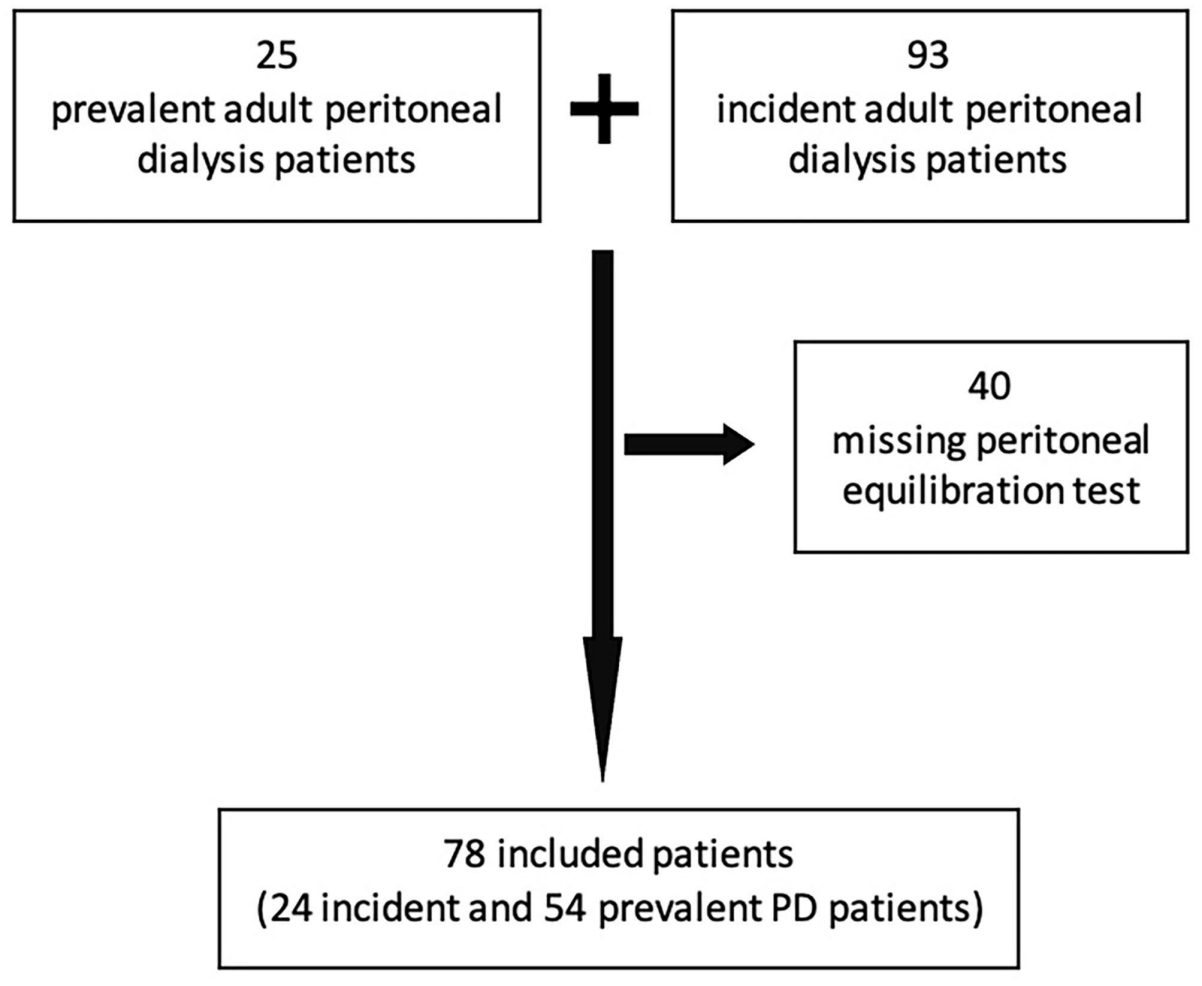
Strobe diagram showing the total number of PD patients assessed for inclusion and included in the study.

A total of 78 patients were included (54 incident, 24 prevalent) out of 118 (Figure 1). Table 1 displays the main baseline characteristics. Diabetes-associated renal disease was the most frequent etiology of renal failure (23%), followed by glomerular disease in 19%, tubulo-interstitial disease in 17%, vascular disease in 12%, and unknown in 19% of the patients. Anuria was present in three patients. The baseline evaluation was performed on the first month of technique (interquartile interval 1–8 months) and these patients had a mean follow-up of 33.9 ± 29.3 months.

**Table 1:** Characterization of the patient population (*n* = 78 patients) and linear univariable regression with peritoneal protein loss.

**Demographics and comorbidities**		**Linear regression with PPL**		
		***B***	**95% CI**	***p***
Age (years)	56 (41–70)	0.048	0.012, 0.085	0.010
Gender, male (%)	65%	1.585	0.187, 2.984	0.027
Follow-up (months)	22 (13–53)			
Charlson comorbidity index	4 (2–6)	0.341	0.062, 0.619	0.017
Diabetes mellitus	26%			n.s.
Cardiovascular disease	27%	1.888	0.401, 3.374	0.014
Systolic blood pressure (mmHg)	138 (122–160)			n.s.
Diastolic blood pressure (mmHg)	87 ± 21			n.s.
Pulse pressure (mmHg)	48 (40–67)	0.037	0.000, 0.074	0.047
**Biochemical evaluation**				
Hemoglobin (g/L)	118 ± 15			n.s.
Blood urea nitrogen (mmol/L)	56.2 ± 17.6			n.s.
Creatinine (μmol/L)	566 (389–796)			n.s.
Glucose (mmol/L)	7.0 (5.9–10.5)			n.s.
Total protein (g/L)	69.3 ± 8.1	−1.183	−2.001, −0.365	<0.001
Albumin (g/L)	39.7 ± 7.2			0.032
High sensitivity C-reactive protein (g/L)	3.7 (1.6–8.1)			n.s.
Interleukin-6 (pg/mL)	5.1 (3.9–9.9)	−1.036	−1.893, −0.089	n.s.
**Dialysis**				
CAPD (vs. cycler)	31%	2.749	1.401, 4.097	<0.001
Icodextrin use	33%			n.s.
**Peritoneal equilibration test and adequacy**				
Time on PD (months)	1 (1–8)			n.s.
D/P creatinine	0.65 ± 0.12	6.595	0.803, 12.387	0.026
Net UF (mL/4 h)	871 ± 259			n.s.
RRF (mL/min/1.73 m^2^)	6.0 (4.1–9.1)			n.s.
Kt/V _urea_ (/week)	2.26 (1.97–2.76)			n.s.
Creatinine clearance (L/1.73/week)	86.5 (65.8–118.6)			n.s.
Peritoneal protein loss (g/24 h)	4.8 (3.8–6.8)			
IL-6 appearance rate (pg/min)*	153.5 (80.7–347.7)	0.002	0.000, 0.004	0.020
**Bioelectrical impedance assessment**				
Soft lean mass/height^2^ (kg/m^2^)**	17.3 ± 2.4	0.455	0.160, 0.750	0.003
Extracellular/total body water (%)	39.4 ± 1.5	53.277	3.981, 102.574	0.035
Extracellular water excess (L)	0.88 ± 0.94	1.365	0.646, 2.083	<0.001

*n.s., non-significant (p-values are shown when <0.2)*.

* IL-6 appearance rate = peritoneal IL-6^*^effluent volume/dwell time in minutes.

** *LBMI reference values: 16.7 kg/m^2^ in males and 13.8 kg/m^2^ in females; for the diagnosis of sarcopenia in PD patients (21)*.

### Univariable Correlation With PPL and Best Multivariable Model

Linear univariable analysis showed positive associations between PPL and (1) small solute transport, as measured by D/P creatinine, (2) body composition, as measured by lean body mass index and overhydration, (3) comorbidities, namely presence of cardiovascular disease or measured by Charlson Comorbidity Index, pulse pressure, older age or male gender (Table 1, right column), (4) performing CAPD vs. cycler. The peritoneal protein loss also correlated positively with dialysate appearance rate of IL-6, but not with serum IL-6. A strong negative correlation was seen between PPL serum albumin and total protein. No significant difference in PPL was found according to icodextrin use or time on PD.

To avoid collinearity, variables with the same biologic meaning were excluded from the multivariable analysis, specifically extracellular water excess was included (excluding extracellular/total body water), and also albumin was entered in the model (ignoring total protein). All the other variables with *p*-value <0.2 shown in Table 1 were included. By linear multivariable analysis, using the backward method, the model with the best adjusted *R*^2^-value showed a significant positive association between PPL and extracellular water excess (95% CI: 4.162–31.854; *p* = 0.012), lean body mass index (95% CI: 1.720–11.219; *p* = 0.008) and performing CAPD (95% CI: 4.375–54.190; *p* = 0.023) were validated. In the best model, cardiovascular disease was considered without attaining statistical significance (Table 2).

**Table 2:** Risk factors associated with peritoneal protein loss.

**Variable**	**PPL**				
	***B***	**β**	**95% CI**	**Tolerance**	**VIF**
CAPD (vs. APD)	1.769	0.265*	(0.284, 3.254)	0.841	1.190
Extracellular water excess	0.789	0.242*	(0.01, 1.567)	0.729	1.371
Cardiovascular disease	1.110	0.150	(−0.559, 2.779)	0.816	1.225
Lean body mass index	0.373	0.293	(0.106, 0.641)	0.950	1.053

*Multivariable linear regression model (backward method), variables included in the model: age, gender, Charlson comorbidity index, cardiovascular disease, pulse pressure, serum IL-6 and albumin levels, CAPD (vs. APD), D/P creatinine, residual kidney function, IL-6 appearance rate, extracellular water excess, peritoneal protein loss and lean body mass*.

* *mean p <0.005*.

*Adjusted R^2^: 0.324, F: 8.773, Durbin Watson: 2.059*.

### Survival Analysis

Overall, 12 patients died (5 deaths due to cardiovascular events, 2 in the context of catastrophic gastrointestinal hemorrhage, 1 death due to Covid-associated pneumonia, 1 death after aspiration pneumonia, other due to vasculitis recurrence and 2 deaths attributed to cachexia). During follow-up. the annual mortality rate of our Unit averaged 6.3%.

In the exploratory survival analysis, no relationship was found between mortality and PPL (HR: 1.020, 95% CI: 0.777–1.339, *p* = 0.886). A univariable positive association was shown with age (HR: 1.077, 95% CI: 1.014–1.144, *p* = 0.016), serum IL-6 concentration (HR: 1.024, 95% CI: 1.006–1.044, *p* = 0.011), pulse pressure (HR: 1.038, 95% CI: 1.002–1.075, *p* = 0.039), overhydration (HR: 2.771, 95% CI: 1.267–6.058, *p* = 0.011) and Charlson Comorbidity Index (HR: 1.331, 95% CI: 1.094–1.620, *p* = 0.004). The presence of cardiovascular disease at baseline assessment showed a trend for worse outcome (HR: 3.101, 95% CI: 0.976–9.854, *p* = 0.055). Higher serum albumin levels were found to be protective (HR: 0.339, 95% CI: 0.117–0.981, *p* = 0.046).

In this early-stage PD population, with globally preserved residual kidney function and lean body mass, an effect of these variables on mortality was not evident. Also, no association with gender, diabetes, dialysate appearance rate of IL-6, CAPD/APD technique or D/P creatinine was found.

Cox regression, conditional backward method, variables included are depicted in Table 3, showed Charlson Comorbidity Index (HR: 1.896, 95% CI: 1.235–2.913, *p* = 0.003), overhydration (HR: 10.034, 95% CI: 1.426–70.587, *p* = 0.021) and lower peritoneal protein loss (HR: 0.576, 95% CI: 0.339–0.978, *p* = 0.041) were predictors for mortality.

**Table 3:** Risk factors predictive of mortality.

**Variable**	**All-cause mortality**		
	**HR**	**95% CI**	***p***
Charlson Comorbidity Index	1.896	(1.235, 2.913)	0.003
Extracellular water excess	10.034	(1.426, 70.587)	0.021
Peritoneal protein loss	0.576	(0.339, 0.978)	0.041

*Multivariable Cox regression model (conditional backward method), variables included in the model: age, gender, Charlson comorbidity index, cardiovascular disease, pulse pressure, serum IL-6 and albumin levels, residual kidney function, extracellular water excess, peritoneal protein loss and lean body mass*.

## Discussion

The purpose of this study was to analyze peritoneal protein loss determinants and to explore prognostic consequences. Most commonly established pathways for higher peritoneal protein leak have been inflammation, in turn associated with peritoneal solute transport rate, and endothelial dysfunction.

In the analyzed cohort, the univariable analysis showed a consistent association of PPL with small solute transport and dialysate appearance rate of IL-6. Davies et al., established that peritoneal protein clearance was a function of local inflammation (as reflected by the product of effective membrane area and local dialysate IL-6 appearance rate) and not systemic inflammation in patients commencing PD (11). Supporting this notion, in our study systemic inflammation, as assessed by serum Il-6, did not predict higher PPL.

The association of PPL and comorbidities, such as measured by Charlson Comorbidity Index, the presence of cardiovascular disease, higher pulse pressure or older age, have been described in several other cohorts, reinforcing the endothelial dysfunction as the origin for such associations (10, 13, 15, 17).

However, multivariable analysis does not support such inferences. The best explicative model enhances overhydration, better nutrition and performing CAPD as best predictors for higher PPL.

As for overhydration, previous studies have established a pathophysiologic mechanism: fluid overload as an important cause for increased venous pressure, causing protein escape from the microcirculation in its venular segment due to venular hydrostatic pressure (16, 17). The magnitude of this increase can be assessed by patients' peritoneal protein loss. Regarding nutritional status, lean body mass index (LBMI) measured by BIA and corrected for body height square has been used as a useful marker (22). Our cohort shows that higher LBMI was independently associated with higher PPL, contradicting the previous viewpoint that higher PPL may cause hypoalbuminemia and malnutrition (1). Previous cohort studies have found similar results, defending that PPL can be compensated by an adequate dietary intake (2, 18) and those patients who have better nutritional state may have more sufficient protein reserves, as well as more active protein metabolism in the peritoneal cavity and lead to more protein loss (18, 23). As for the relation of peritoneal protein leak with PD technique, this issue has also revealed to be a controversial topic. Kathuria et al., back in 1997, found no difference in nocturnal intermittent PD vs. CAPD, in terms of peritoneal protein leakage (24). In our cohort CAPD was associated with higher PPL. This comes in accordance to an interventional study, done by Cueto-Manzano et al., demonstrating lower PPL with short dwell-time periods and extended dry periods (25). Despite the controversy, APD could be a more feasible option in nephrotic patients, in order to decrease PPL, which could aggravate the clinical features in presence of the high proteinuria (opinion).

In the exploratory survival analysis, higher mortality was higher in overhydrated patients, with higher Charlson Comorbidity Index, but not peritoneal protein losses. The importance of fluid overload explains the association between peritoneal protein clearance and mortality reported in some epidemiologic studies and refutes assumptions on a possible role of endothelial dysfunction or inflammation (17, 26).

The present study presents with a number of limitations. It was a single-center cross study with a limited number of participants, mostly in early stage of PD exposure. The low number of deaths and the lengthiness of the study could be a limitation for the survival analysis. Strengths of this study rely on the use of measured total protein for 24 h peritoneal protein loss (instead of calculation) and second, the early stage of PD exposure can also be seen as a strength, as it enables insight in normal peritoneal physiology. Third, the use of bioimpedance to evaluate the link between PPL and body composition as an important evaluation tool, allowing the adjustment for multiple confounding covariates. It is recommendable to confirm these results in larger series.

This study illustrates the importance of overhydration, as a strong predictor of PPL, overpowering variables previously reported as determinants of PPL, namely clinical correlates of endothelial dysfunction or local inflammation. Also, survival analysis demonstrates the importance of overhydration as a strong prognostic factor. Peritoneal protein losses were not associated with malnutrition or higher mortality, emphasizing the importance of volume overload control, amenable by adjusted dialysis prescription, diuretic and water intake restriction.

## Data Availability Statement

The raw data supporting the conclusions of this article will be made available by the authors, without undue reservation.

## Ethics Statement

The studies involving human participants were reviewed and approved by Ethics Committee of the Centro Hospitalar Universitário do Algarve (UAIF 192/2019). The patients/participants provided their written informed consent to participate in this study.

## Author Contributions

AM: responsible for the investigational project, elaboration of the manuscript, and statistical analyses. RC and BR: responsible for clinical care and data collection. BR, MTF, MF, and JB: responsible for biological samples care and analysis. AS and AR: responsible for the investigational project and for reviewing the manuscript. All authors contributed to the article and approved the submitted version.

## Funding

This work was funded by the grant numbers UIDB/00215/2020, UIDP/00215/2020, and LA/P/0064/2020 respectively.

## Conflict of Interest

The authors declare that the research was conducted in the absence of any commercial or financial relationships that could be construed as a potential conflict of interest.

## Publisher's Note

All claims expressed in this article are solely those of the authors and do not necessarily represent those of their affiliated organizations, or those of the publisher, the editors and the reviewers. Any product that may be evaluated in this article, or claim that may be made by its manufacturer, is not guaranteed or endorsed by the publisher.

## Footnotes

^1^https://www.mdcalc.com/charlson-comorbidity-index-cci (accessed during the study time period).

## References

[1] Dulaney JT Hatch FE Jr. Peritoneal dialysis and loss of proteins: a review. Kidney Int. (1984) 26:253–62. 10.1038/ki.1984.167 6392691

[2] Malho Guedes A. Peritoneal protein loss, leakage or clearance in peritoneal dialysis, where do we stand? Perit Dial Int. (2019) 39:201–9. 10.3747/pdi.2018.00138 31088933

[3] Rajakaruna G Caplin B Davenport A. Peritoneal protein clearance rather than faster transport status determines outcomes in peritoneal dialysis patients. Perit Dial Int. (2015) 35:216–21. 10.3747/pdi.2013.00217 25082839 PMC4406317

[4] Lu W Pang WF Jin L Li H Chow KM Kwan BC. Peritoneal protein clearance predicts mortality in peritoneal dialysis patients. Clin Exp Nephrol. (2019) 23:551–60. 10.1007/s10157-018-1677-9 30506285

[5] Perl J Huckvale K Chellar M John B Davies SJ. Peritoneal protein clearance and not peritoneal membrane transport status predicts survival in a contemporary cohort of peritoneal dialysis patients. Clin J Am Soc Nephrol. (2009) 4:1201. 10.2215/CJN.01910309 19478100 PMC2709517

[6] Heaf JG Sarac S Afzal S. A high peritoneal large pore fluid flux causes hypoalbuminemia and is a risk factor for death in peritoneal dialysis patients. Nephrol Dial Transplant. (2005) 20:2194–201. 10.1093/ndt/gfi008 16030031

[7] Pérez-Fontán M Rodríguez-Carmona A Barreda D López Muñiz A Blanco Castro N García Falcón T. Peritoneal protein transport during the baseline peritoneal equilibration test is an accurate predictor of the outcome of peritoneal dialysis patients. Nephron Clin Pract. (2010) 116:c104–13. 10.1159/000314659 20502046

[8] Kang DH Yoon KI Choi KB Lee R Lee HY Han DS. Relationship of peritoneal membrane transport characteristics to the nutritional status in CAPD patients. Nephrol Dial Transplant. (1999) 14:1715–22. 10.1093/ndt/14.7.1715 10435882

[9] Tang Y Zhong H Diao Y Qin M Zhou X. Peritoneal transport rate, systemic inflammation, and residual renal function determine peritoneal protein clearance in continuous ambulatory peritoneal dialysis patients. Int Urol Nephrol. (2014) 46:2215–9. 10.1007/s11255-014-0744-8 24894487

[10] Chang TI Kang EW Lee YK Shin SK. Higher peritoneal protein clearance as a risk factor for cardiovascular disease in peritoneal dialysis patient. PLoS ONE. (2013) 8:e56223. 10.1371/journal.pone.0056223 23418538 PMC3571965

[11] Yu Z Lambie M Chess J Williams A Do J-Y Topley N Davies SJ. Peritoneal protein clearance is a function of local inflammation and membrane area whereas systemic inflammation and comorbidity predict survival of incident peritoneal dialysis patients. Front Physiol. (2019) 10:105. 10.3389/fphys.2019.00105 30833904 PMC6387967

[12] Zemel D Koomen GCM Hart AAM ten Berge RJM Struijk DG Krediet RT. Relationship of TNFat interleukin-6 and prostaglandins to peritoneal permeability for macromolecules during longitudinal follow-up of peritonitis in continuous ambulatory peritoneal dialysis. J Lab Clin Med. (1993) 122:686–96. 10.1159/000187340 8245688

[13] Sanchez-Villanueva R Bajo A del Peso G Fernandez-Reyes González E Romero S. Higher daily peritoneal protein clearance when initiating peritoneal dialysis is independently associated with peripheral arterial disease (PAD): a possible new marker of systemic endothelial dysfunction? Nephrol Dial Transplant. (2009) 24:1009–14. 10.1093/ndt/gfn595 18997161

[14] Balafa O Halbesma N Struijk DG Dekker FW Krediet RT. Peritoneal albumin and protein losses do not predict outcome in peritoneal dialysis patients. Clin J Am Soc Nephrol. (2011) 6:561–6. 10.2215/CJN.05540610 21071518 PMC3082414

[15] Elsurer R Afsar B Sezer S Ozdemir FN Haberal M. Peritoneal albumin leakage: 2-year prospective cardiovascular event occurrence and patient survival analysis. Nephrology. (2009) 14:712–5. 10.1111/j.1440-1797.2009.01103.x 20025678

[16] Krediet RT Yoowannakul S Harris L Davenport A. Relationships between peritoneal protein clearance and parameters of fluid status agree with clinical observations in other diseases that venous congestion increases microvascular protein escape. Perit Dial Int. (2019) 39:155–62. 10.3747/pdi.2018.00016 30661003

[17] Malho Guedes A Marques R Domingos AT Silva AP Bernardo I Neves PL Rodrigues A Krediet RT. Overhydrattion may be the missing link between peritoneal protein clearance and mortality. Nephron. (2021) 145:474–80. 10.1159/000516531 34130276

[18] Do JY Kim AY Kang SH. Peritoneal protein loss is not associated with sarcopenia in peritoneal dialysis patients. Front Med. (2021) 8:653807. 10.3389/fmed.2021.653807 34336874 PMC8316630

[19] Ohashi Y Joki N Yamazaki K Kawamura T Tai R Oguchi H. Changes in the fluid volume balance between intra- and extracellular water in a sample of Japanese adults aged 15-88 yr old: a cross-sectional study. Am J Physiol. (2018) 314:F614–22. 10.1152/ajprenal.00477.2017 29212765

[20] AAT Bioquest, Inc. Quest Graph™ Four Parameter Logistic (4PL) Curve Calculator. Available online at: https://www.aatbio.com/tools/four-parameter-logistic-4pl-curve-regression-online-calculator (accessed August 12, 2021).

[21] Xu X Yang Z Ma T Li Z Chen Y Zheng Y. The cut-off values of handgrip strength and lean mass index for sarcopenia among patients on peritoneal dialysis. Nutr Metab. (2020) 17:84. 10.1186/s12986-020-00506-3 33062032 PMC7542899

[22] Desmeules S Levesque R Jaussent I Leray-Moragues H Chalabi L Canaud B. Creatinine index and lean body mass are excellent predictors of long-term survival in haemodiafiltration patients. Nephrol Dial Transplant. (2004) 19:1182–9. 10.1093/ndt/gfh016 14993499

[23] Fan J Ye H Zhang X Cao P Guo Q Mao H. Association of lean body mass index and peritoneal protein clearance in peritoneal dialysis patients. Kidney Blood Press Res. (2019) 44:94–102. 10.1159/000498841 30808849

[24] Kathuria P Moore HL Khanna R Twardowski ZJ Goel S Nolph KD. Effect of dialysis modality and membrane transport characteristics on dialysate protein losses of patients on peritoneal dialysis. Perit Dial Int. (1997) 17:449–54. 10.1177/089686089701700507 9358526

[25] Cueto-Manzano AM Gamba G Correa-Rotter R. Peritoneal protein loss in patients with high peritoneal permeability: comparison between continuous ambulatory peritoneal dialysis and daytime intermittent peritoneal dialysis. Arch Med Res. (2001) 32:197–201. 10.1016/S0188-4409(01)00271-5 11395184

[26] Krediet RT Barreto DL van Diepen AT. Assessment of the size selectivity of peritoneal permeability by the restriction coefficient to protein transport. Perit Dial Int. (2022) 1:8968608221075102. 10.1177/08968608221075102 35102776

